# Low-Energy Electron Inelastic Mean Free Path of Graphene Measured by a Time-of-Flight Spectrometer

**DOI:** 10.3390/nano11092435

**Published:** 2021-09-18

**Authors:** Ivo Konvalina, Benjamin Daniel, Martin Zouhar, Aleš Paták, Ilona Müllerová, Luděk Frank, Jakub Piňos, Lukáš Průcha, Tomáš Radlička, Wolfgang S. M. Werner, Eliška Materna Mikmeková

**Affiliations:** 1 Institute of Scientific Instruments of the Czech Academy of Sciences, Královopolská 147, 612 64 Brno, Czech Republic; bdaniel@isibrno.cz (B.D.); zouharm@isibrno.cz (M.Z.); patak@isibrno.cz (A.P.); ilona.mullerova@isibrno.cz (I.M.); ludek@isibrno.cz (L.F.); pinos@isibrno.cz (J.P.); prucha@isibrno.cz (L.P.); radlicka@isibrno.cz (T.R.); eliska@isibrno.cz (E.M.M.); 2 Institute of Applied Physics, Vienna University of Technology, Wiedner Hauptstraße 8–10/E134, 1040 Vienna, Austria; wolfgang.werner@tuwien.ac.at

**Keywords:** time-of-flight spectrometer, inelastic mean free path, density-functional theory, many-body perturbation theory, energy-loss spectrum, density of states, band structure, graphene

## Abstract

The detailed examination of electron scattering in solids is of crucial importance for the theory of solid-state physics, as well as for the development and diagnostics of novel materials, particularly those for micro- and nanoelectronics. Among others, an important parameter of electron scattering is the inelastic mean free path (IMFP) of electrons both in bulk materials and in thin films, including 2D crystals. The amount of IMFP data available is still not sufficient, especially for very slow electrons and for 2D crystals. This situation motivated the present study, which summarizes pilot experiments for graphene on a new device intended to acquire electron energy-loss spectra (EELS) for low landing energies. Thanks to its unique properties, such as electrical conductivity and transparency, graphene is an ideal candidate for study at very low energies in the transmission mode of an electron microscope. The EELS are acquired by means of the very low-energy electron microspectroscopy of 2D crystals, using a dedicated ultra-high vacuum scanning low-energy electron microscope equipped with a time-of-flight (ToF) velocity analyzer. In order to verify our pilot results, we also simulate the EELS by means of density functional theory (DFT) and the many-body perturbation theory. Additional DFT calculations, providing both the total density of states and the band structure, illustrate the graphene loss features. We utilize the experimental EELS data to derive IMFP values using the so-called log-ratio method.

## 1. Introduction

Further technological progress and innovations, e.g., in the field of semi-conductors, is a current challenge for the industry. This motivates the search for novel materials, which in turn makes the requirements for techniques of analysis higher. Layered thin materials, constructed by “stacking” 2D sheets on top of each other, represent a class of promising materials. Detailed knowledge of the interaction of electrons with materials is of prime importance for the development of new materials for next-generation electronic devices. This makes analysis techniques using electrons as probes a natural choice for the analysis of such materials.

**Figure 1:**
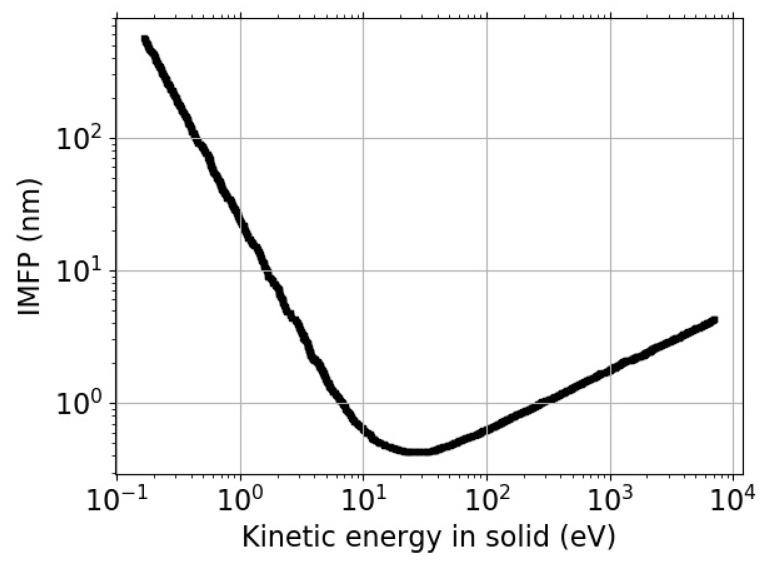
Universal curve of IMFP versus kinetic energy derived for amorphous materials [2,3].

The inelastic mean free path (IMFP) can be defined as the mean distance between two subsequent inelastic scattering events (measured along its trajectory). Such energy-loss processes imply a transfer of momentum and energy to the solid-state electrons [1]. The electron IMFP in solids is one of the most important parameters of the electron and depends on the kinetic energy of electrons. The relationship between the two is often described by a so-called “universal curve”, shown in Figure 1, interpolating the experimental data points mainly for the amorphous or polycrystalline materials.

Any inhomogeneity or crystallinity [4] of the material can cause deviations from the “universal curve”. Certain surface-bound phenomena affect the experimentally established IMFP values. Its significance grows with diminishing thickness of the samples, especially of thin films and 2D materials. Hence, the validity of the “universal curve” is not guaranteed when one switches from bulk samples—for which it was originally obtained—to 2D crystals [5]. Additionally, an overwhelming majority of the published data used to form the “universal curve” were obtained using fast electrons, while the micro- and nano-electronic applications of materials utilize electrons of several orders of magnitude slower.

For example, the transmissivity of very slow electrons through graphene layers decreases when the kinetic energy is decreased [6] although an increase would be expected when only taking into account the IMFP “universal curve”. This makes the determination of the IMFP, especially for ultra-thin samples and 2D materials—such as graphene—in low-energy regimes, of high interest and importance.

Though one can use optical absorption data to determine the IMFP, employing electron energy-loss spectra (EELS) is more frequent. These data are often utilized within a so-called “dielectric function” formalism, fitting a certain model of the dielectric function ε to the spectra; the choice of model is important, as it may affect the observables [7].

Theory is presently commonly used in tabulations of the key electron transport parameters, such as IMFP, attenuation lengths, and the mean escape depths of electrons. The predictions are rather in fair agreement with the experimental data for energies, *E*, of a few hundred electron volts, say, *E* > 200 eV, but exhibit considerable differences at lower electron energy. With progress in the instrumentation, some of the older measurements, on which the theory is based, can be improved by the current state of the art. As a result, the currently used results may be corrected and using the “old” ones may contribute to the aforementioned discrepancies. Another reason is the application of approximations that are invalid for low-energy electrons [8]. Moreover, for low electron energies (comparable with energy band gaps), the electron inelastic processes are damped, possibly leading to a modulation of the IMFP’s dependence on energy or even on the band structure of the materials. Recently, promising progress has been made in calculation and measurements from X-ray absorption spectra [9]. Nevertheless, the experimental values of the IMFP in the low-energy range are rarely reported [10,11,12].

The above overview makes it clear that obtaining IMFP values in the low-energy range for 2D materials, e.g., for graphene, is a challenging task. There are no reliable ways to predict the IMFP values in this situation if one disregards ab initio modeling. The reason lies in a specific scattering scenario different from that in the bulk. Particularly, surface excitation phenomena occur on both sides of the 2D samples [13,14]. As a result, the electron energy dependence of IMFP values can differ from that of 3D materials, in both the low and the high electron energy ranges. We expect the measured values to be sensitive to surface contamination as it can, e.g., easily increase the thickness of the 2D sample by a factor of two [15]. Up to now, the IMFP (or, precisely, the attenuation length) values in the case of very thin films and/or 2D materials were determined by using electron microscopy methods for samples with different numbers of mono-atomic layers [16,17], by Auger electron spectroscopy [16], photoelectron spectroscopy [18,19,20], and reflection electron energy-loss spectroscopy (REELS) [21], mostly providing a few values only. In addition, the spin-resolved IMFP of slow electrons in Fe has also been studied [22].

The anisotropy of electron attenuation in 3D crystals brings about an additional uncertainty in the determination of IMFP. This effect, already observed in angle-resolved photoelectron spectroscopy, has been interpreted simply as an enhanced electron channeling process along the atomic rows [23]. A more detailed theoretical explanation considers multiple electron scattering. One can expect analogous anisotropy in the case of 2D crystals as well, not only in directions perpendicular to the atomic plane but also in different directions within the plane, responsible for the azimuthal anisotropy of emitted and transmitted intensities.

Theoretical approaches for IMFPs involve predictive formulas such as TPP-2M [24], G1 [25], or Bethe formula [26] (again, all three are valid for amorphous materials) and they may also involve ab initio density functional theory (DFT). Calculations of the IMFP from optical data and electron spectroscopy data in the energy range 50–2000 eV were reported in the past [27,28,29].

Ideally, experimental EELS data can be collected on a fully homogeneous single crystal, but it is usually necessary to examine materials that are heterogeneous in structure or composition, or both. In this case, spatially resolved data must be collected using a microscopic technique. The two available alternatives include spectromicroscopy, i.e., the energy-sensitive filtration of the complete micrographs using a position-sensitive filter, or microspectroscopy, i.e., the consecutive single channel filtration of signals from image pixels. In both cases, three-dimensional x–y-energy image data are collected and subsequently processed along the energy axis for every location.

The spectromicroscopic regime is typical for energy filtered transmission electron microscopy (EFTEM) instruments [30], in which samples of a micrometer or smaller thickness are examined with primary electrons, as a rule, in the energy range of tens or hundreds of keV, and energy losses up to tens or hundreds of eV are detected. Energy filtration is performed below the sample on transmitted electrons that are not elastically scattered far off the optical axis. The scattering data for slow electrons cannot be obtained using this mode. The microspectroscopy technique is often available in scanning electron microscopes (SEM) equipped with energy analyzers attached aside the specimen stage, usually with a rather limited angular range of acceptance of the backscattered electron (BSE) emission. The energy analyzers, more precisely the velocity analyzers employed here, are generally of the dispersion type, such as the cylindrical mirror analyzer or the concentric hemispherical analyzer [31]. The electron transmissivity of the analyzer input, governing the signal-to-noise ratio in the spectroscopic data, is usually rather low in this alternative, because the desired energy resolution requires to restrict the angular acceptance of the filter and the allowed entry pupil is limited in size as well. Moreover, data based on BSE are generally burdened with locally varying information depth dependent on material composition and the crystalline structure and orientation.

The most promising technique to study 2D crystals is microspectroscopy based on a scanning transmission electron microscope (STEM) instrument employing the cathode lens principle for the retardation of the primary electrons to an arbitrarily low impact energy [32] so that scattering of very slow electrons in the sample can be examined. Velocity filtration is performed below the sample and a variety of analyzers can be introduced, such as dispersion types, for example. This combination can work for very slow scattered electrons with the sample biased to high negative potential and the filter input at ground potential. The analyzer is then passed by fast electrons, resulting in the resolving power, inversely proportional to their energy, being rather low [33]. Moreover, the dispersion analyzers allow one to accept a small field of view only as a source of electrons under analysis. An analyzer of the time-of-flight (ToF) type with a drift tube situated below the sample stage can be completely held on the sample potential, hence analyzing non-accelerated transmitted electrons with high resolution. However, in ToF analysis, a requirement, among others, is to chop the primary electron beam to pulses as short as possible.

Because of the aforementioned issues, we decided to measure the EELS of very slow electrons transmitted through 2D crystals (or ultrathin films) using a dedicated ultra-high vacuum (UHV) scanning low-energy electron microscope (SLEEM) instrument equipped with a cathode lens and the time-of-flight velocity analyzer situated below the sample.

We select graphene, a representative of 2D materials, to demonstrate the capabilities of our device. Graphene has been an intensively studied material since its discovery. Its EELS spectrum contains two dominant loss features interpreted as a π-plasmon and (π + σ)-plasmon occurring at approximately 4 eV and 13.5 eV, respectively. These values correspond to momentum transfers (MT) close to zero.

## 2. Materials and Methods

Our system allows one to study free-standing samples without a carrier; hence, the measured spectra do not show the substrate-related peak and other effects. This removes both effects related to the buffer layer and the substrate that can significantly affect the electron effective attenuation length (EAL). The EAL for free-standing graphene can be increased by approximately tens of percent [34].

A rather recent trend is to complement the experimental results by theoretical simulations that may provide some insight into and interpretation of the processes that lead to the observed results. The theoretical results can also hint at what to expect from the measurements. The simulation methods include, e.g., MC or ab initio methods. They allow one to study the extent of surface effects on the inelastic scattering of a Si film [35,36].

The experimental EELS is what we used to calculate the effective IMFP. We also studied the sample using theoretical simulations by means of DFT and many-body perturbation theory (MBPT). The theoretical results include the band structure, density of states (DOS) and momentum resolved EELS, with the last one being calculated within the dielectric function formalism using MBPT. These simulations corroborate both the experimental results and related data processing.

**Figure 2:**
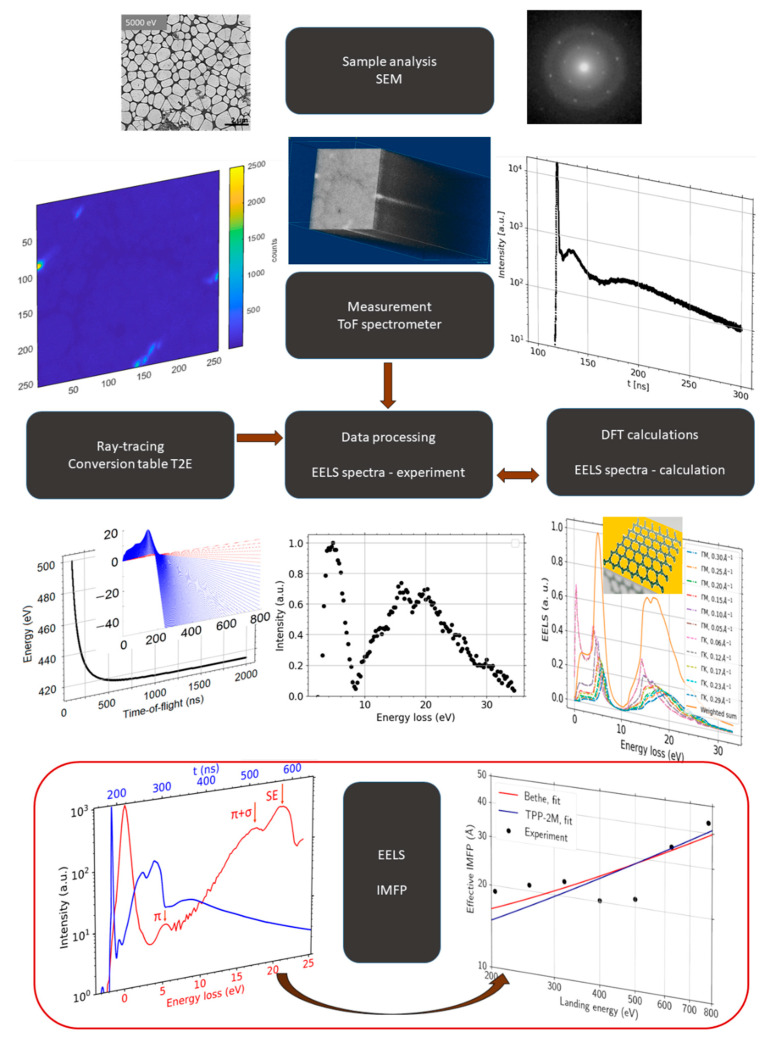
An overview diagram showing the individual steps of the sample analysis, starting with the sample analysis (**top**), followed by ToF measurement and processing based on theoretical ray-tracing up to the both ZLP and baseline corrected spectra. Ab initio simulations provide an alternative data input to confirm the measured results. Finally, the ToF measurements at different landing energies allow to determine the energy dependence of the IMFP (**bottom**).

Figure 2 is an overview diagram that shows sample analysis, measurement, data processing, ab initio simulation, and determination of the IMFP vs. energy dependence.

This section describes the device, data processing methods and theoretical calculations. Section 3 presents the experimental and theoretical results, including the effective IMFP obtained from the experimental EELS data. The results are followed by Section 4, which contains their discussion. We end the paper with conclusions in Section 5.

### 2.1. SLEEM/ToF Device

**Figure 3:**
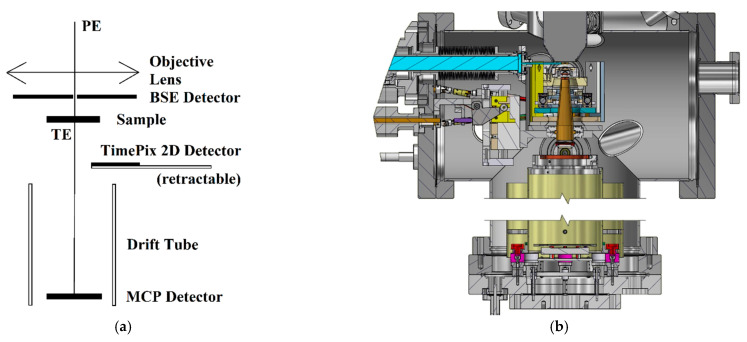
Schematic of the UHV SLEEM/ToF system with the ToF spectrometer installed under the sample for TEs detection (**a**). Section of a 3D model of sample chamber and ToF spectrometer (**b**).

The UHV SLEEM/ToF system [37], developed at the Institute of Scientific Instruments (ISI), is shown in Figure 3.

The spectrometer can operate as a standard SEM (giving an image of the sample) with an ability to yield low-energy-loss data from a specific area of the sample. The microscope is equipped with an electron gun providing 5 keV primary beam energy, developed by Delong Instruments, Inc. [38], an in-house built specimen stage with biased specimen holder enabling the cathode lens (CL) mode in the SLEEM [32], and several electron detectors for secondary, backscattered, and transmitted electrons. The CL mode increases the resolution of the objective lens for low landing energies [39].

We designed and assembled a ToF spectrometer for the detection of very slow transmitted electrons from the studied samples. The ToF spectrometer consists of two focusing electrodes: a transport tube, a 620 mm long drift tube surrounded by an electromagnetic shielding envelope, and a multi-channel plate (MCP) detector. A newly added “pulsing” operation mode allows for the measurement of ToF spectra [40] of electrons transmitted through the sample.

A typical limit of energy resolution for a simple (field free) ToF spectrometer depends on electron energy, the length of the flight path and time resolution. The time resolution is a combination of contributions from the duration of the electron pulse (1–4 ns), the detector resolution (approximately 0.2 ns) and the angular beam spread, leading to variations in the flight path length (depending on electrode settings, typically 0.2 ns). The best time resolution measured is 0.7 ns full width at half maximum (FWHM), which is obtained using 1 ns pulse width. Usually, 2 ns pulses are used, which decreases the time resolution to 1.5 ns. An additional resolution limit is the energy spread of 0.6 eV, which is the limiting contribution below 50 eV. Applying a monochromator would result in much better resolution.

In order to implement the pulse mode, the commercial electronics provided with the MCP detector [41] was complemented by other components, including a pulse generator deflecting the primary electron beam. The software supplied to the MCP detector, integrated in the ToF spectrometer, records the timestamps of detections for each electron pulse sent towards a given pixel of the sample. These timestamps, combined with signals from the pulse generator, allow for the reconstruction of the ToF spectra and processing, described in detail in the subsequent section.

The aim of the development of the ToF spectrometer was to achieve the measurement of the time-of-flight with a resolution below 1 ns. The calculated energy resolution is limited by the initial temporal spread of the pulse. We actually achieved a value of 0.7 ns with the electron gun based on a Schottky cathode.

The energy resolution [42] of the ToF spectrometer can be approximated by an equation:

(1) $$dE=\frac{2\sqrt{2}}{D\sqrt{m}}E^{3/2}dt\;$$

where *dE* denotes the energy uncertainty, *D* is equal to the drift tube length, *m* stands for the electron mass, *E* is the electron energy and *dt* denotes the uncertainty in the flight time. For a time-of-flight equal to 150 ns, the energy resolution of the ToF spectrometer is approximately 0.5 eV for the landing energy of 50 eV, while for the landing energy of 25 eV, the energy resolution is 0.17 eV. This value is sufficient for the study of energy spectra of the transmitted electrons and the secondary electrons excited during the passage of the beam.

### 2.2. Data Processing

In-house-made Matlab + C tracing scripts allow one to optimize the voltages set on the individual electrodes for a given landing energy, *E_L_*, and provide the conversion table, i.e., theoretical dependence, *t(E)*, of the ToF on energy, *E*, of the system with these specific settings. These scripts help to find an optimum setup for each measurement.

**Figure 4:**
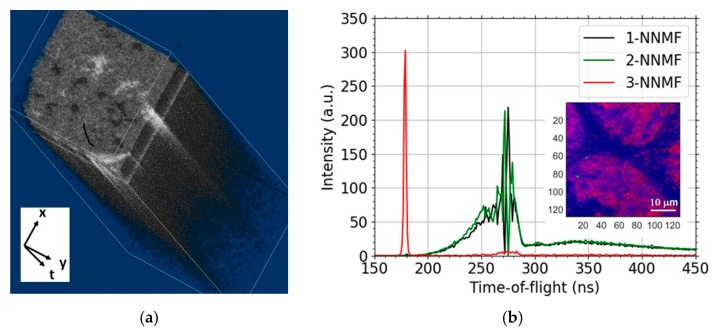
Transmission image of the graphene sample taken by the MCP detector and the measured ToF spectra. Visualization of the 3D hyperspectral data, the pixel sliced time-domain histogram collected over all frames (**a**). The non-negative matrix factorization (NNMF) of spectra corresponding to another field of view (**b**).

The measurement consists of the acquisition of the timestamps corresponding to the individual events, pixel switch *t_switch_*, trigger pulse sent *t_pulse_* and detection event *t_detect_*. These raw data are converted in true hyper-spectral imaging data of ToF “times” (*t_detect_* − *t_pulse_*). Line and frame switches are identified from regular patterns in the pixel switch signals, which allows one to assign a position to each pixel. Thence, we acquire a collection of ToF values corresponding to a given pixel in a given scan of the selected window, i.e., an image frame. In other words, ToF spectra for each set of the three coordinates—scan/frame no. and two pixel coordinates. We visualize the hyper-spectral data in Figure 4a, displaying density of counts in the time-domain histogram for each pixel collected over all frames. Consider a slice corresponding to a given time bin. We see that the pixels in the slice may contain areas of significantly different densities of detections (higher values are lighter in the image), i.e., the transmissivity, as recorded by the MCP.

The fact that the ToF spectra are pixel-resolved allows us to apply a decomposition, e.g., principal component analysis, independent component analysis and/or non-negative matrix factorization (NNMF). Such a decomposition is an attempt to reduce the spectral dimensions. This allows the data to be displayed as three two-dimensional images. A result of the NMMF procedure is presented in Figure 4b, with each of the three data-sets plotted using a specific color. The resulting images are adjusted for good visibility (each component is scaled to make full use of the range of the vertical axis), and thus the images do not reflect the actual relative size of components, but allow one to obtain a reasonably good overview at a single glance.

**Figure 5:**
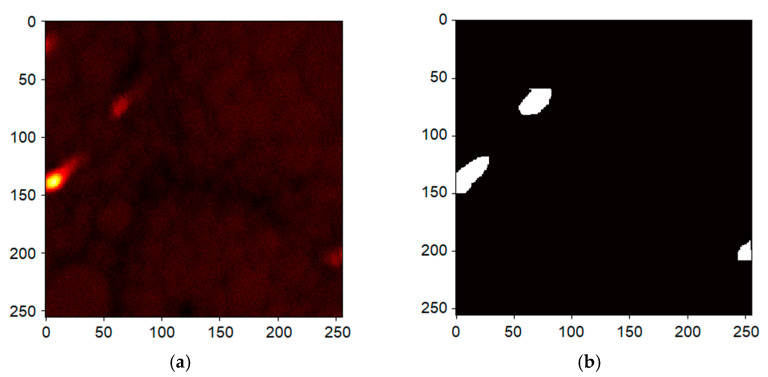
Heat-map of counts of a 500 eV landing energy measurement (sum over all frames). The high intensity regions are interpreted as holes (**a**) providing a mask (**b**); pixels in the white regions are excluded from the cumulative data used to provide the energy-domain histogram.

The total count per pixel collected over all complete frames is visualized in Figure 5a, and it displays variations among pixels already visible in Figure 4a. We interpret the significantly higher count regions as a lower quality sample, e.g., holes, and we eliminate them from the further processing by applying a mask displayed in Figure 5b; the mask makes sure the white regions are ignored.

If there are holes in the sample, the corresponding spectra may provide a “dark signal” (different from the zero-loss peak) to be subtracted from the sample-pixel spectra. The mask-selected data are collected, corrected to an optional baseline, representing the noise of constant value, dark data subtraction and a time shift to adjust, e.g., for propagation in wires, and converted to the energy-domain using the theoretical conversion table. There are two forms of conversion available. First, the corrected time-domain histogram is converted into the energy-domain histogram involving Jacobian of the mapping. Second, we convert the as-determined individual ToF values to energy directly and then create an energy-domain histogram without the constant baseline correction. Let us note that the constant noise is due to the ion pumps with a marginal dark count contribution to the MCP detector (typically tens of counts per second).

The energy-domain histogram prepared in this way can be further processed as follows. The zero-loss peak (ZLP) is fit to an appropriate shape. We use the Gaussian function for the ZLP fit but other forms are also available. The fit of the ZLP can be improved by providing automatic and/or custom weights. We can manually select the energy range of data used for the fit and increase the weights of the data-points at the top of the peak and at the right-most part of the selected region. This improves the quality of the low-loss part of the energy spectra after removal of the ZLP.

Additionally, if the need arises, the spectra can be deconvolved using the ZLP-fit data via the Richardson–Lucy [43,44] algorithm. This allows for some features apparently merged with the ZLP signal to stand out clearly. The ZLP of the deconvolved spectra can be re-fit and subtracted. In any case, we try to keep the processing of measured spectra to a reasonable minimum extent due to the “tricky” nature of the ZLP-fit (different curves, symmetric vs. asymmetric).

Finally, one can remove the background using a non-constant baseline containing the asymmetric least square algorithm [45,46] (semi-automatic). Alternate simpler background removal consists of providing the spectrum data-points used to construct straight-line segments composing an alternate baseline that is subtracted. This final result is suitable for comparison with the EELS spectra.

We can use the baseline/background corrected spectra to estimate the effective IMFP via the log-ratio method [47]. It is the simplest method to determine an effective IMFP λ_IMFP_ value from energy-loss spectra measured at a given landing energy *E_L_*,

(2) $$\frac{d}{\lambda_{IMFP}\;\left(E_{L}\right)}=ln\frac{S_{total}}{S_{ZLP}}$$

where

(3) $$S_{range}=\int_{range}^{}dE\;I\left(E\right)$$

and *d* denotes the sample thickness. The “range” in Equation (3) is selected to be the either “total” spectrum (or its relevant portion) or the “ZLP” part in Equation (2). The area of the ZLP can be obtained in two ways. First, by selecting the energy-loss range of the ZLP in the spectrum directly. The suitable boundary values—lower limit (negative), where the ZLP sufficiently decreases, and the first minimum in intensity following the ZLP that separates the right side of the ZLP from the rest of the spectra—for the integration are provided manually. Second, via the area under a theoretical fit of the ZLP. Thus, the obtained values of the IMFP are “effective” in the sense that they depend on the true aperture size; in other words, the acceptance polar angle which translates to detected values of the MT. Since we mostly restrict the measured EELS to the plasmon excitation range, to be correct, we should refer to the determined IMFP as an “IMFP for plasmon losses”. We omit this additional denotation in the following for the sake of brevity.

Custom Matlab and Python scripts [48,49,50,51] implement the above-described processing.

### 2.3. DFT Calculations

We also studied the infinite free-standing monolayer graphene by means of density-functional theory, employing the Quantum Espresso software [52]. The DFT calculations were performed using the local-density approximation (LDA). The optimized norm-conserving Vanderbilt pseudopotential [53] was utilized. The object was described by a hexagonal two-atom cell. The cut-off of the plane-wave basis set is 90 Ry. The relaxed value of the in-plane lattice constant was *a* = 2.449 Å, which agrees well with experiments, namely (2.45 ± 0.04) Å for graphene on Ir(111) [54] and (2.4589 ± 0.0005) Å for graphite [55], while the height of the vacuum along the z-direction was equal to 38 Bohr. The self-consistent and non-self-consistent calculations used 40 × 40 × 1 and 90 × 90 × 1 sampling of the reciprocal space, respectively. The individual k-points were generated using the Monkhorst–Pack algorithm. The Savitzky–Golay filter [49] was used to smooth out tiny oscillations in the total density of states, appearing due to the finite number of k-points, without affecting relevant features.

We also performed the calculation of momentum-resolved EELS spectra using MBPT on top of the DFT, as implemented in the Yambo code [56]. The calculations utilized the random phase approximations (RPA) and the adiabatic-LDA (ALDA) with the inclusion of local-fields effects. Furthermore, because the system under study was a 2D material, both the random integration method and cut-off Coulomb potentials were applied. Convergence tests led to the following values of the relevant parameters of the calculations. The number of k-points in the grid was 90 × 90 × 1, the super-cell size in the perpendicular direction to graphene was 55 Bohr, and the energy cut-off for expanding the wave-functions (FFTGvecs) was 50 Ry. The maximal number of bands entering in the sum over states in the RPA response function (BndsRnXd) converged at 70. Energy cut-off in the screening (NGSBlkXd) and XC-kernel size (FxcGRLc) were converged simultaneously to the final value of 2928 mRy. We used 500 energy steps (ETStpsXd) and accelerated the calculations (GTemKind set to ‘BG’).

## 3. Results

**Figure 6:**
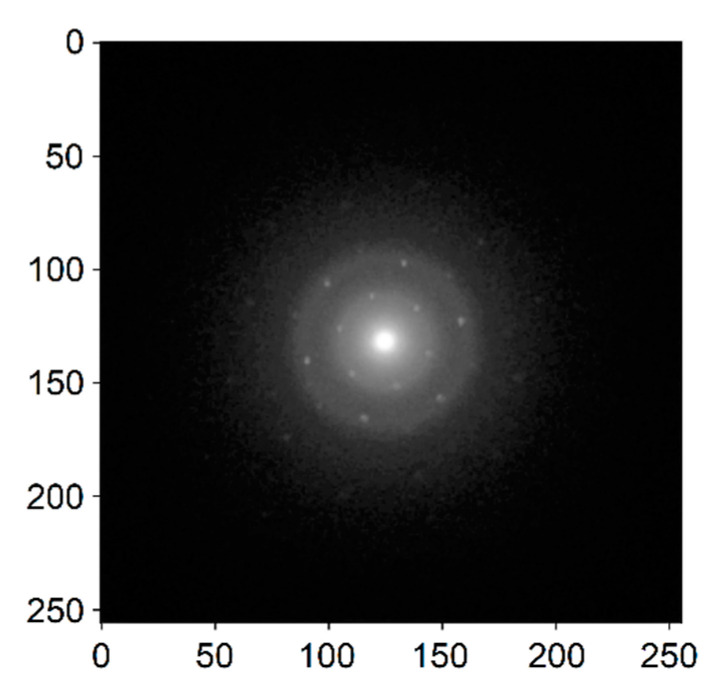
Diffraction patterns of a monolayer graphene obtained with a pixelated detector in Helios SEM; *E_P_* = 30 keV, *I_P_* = 13 pA, 256 × 256 px, camera length = 36.7 mm.

In this section, we present the results acquired by the ToF spectrometer on a commercially available single layer graphene sample by the Ted Pella company [57] and compare them with existing literature to illustrate the capabilities of the device. In order to verify the origin of the measured spectra from single-layer graphene, we used a method based on the comparison of diffraction data [58]—the number of layers was determined from the intensity of the diffraction spots, namely differences in the intensity between the first- and higher-order diffraction spots. An example diffractogram for one-layer graphene obtained from Helios SEM (Thermo Fisher Scientific, Brno, Czech Republic) using pixelated STEM detector T-pix for transmitted electrons is shown in Figure 6. We can see that the intensity of the spots is practically identical within each diffraction order. The image has a six-fold symmetric distribution of the spots. This implies that the corresponding region of the sample is most likely a mono-layer graphene.

**Figure 7:**
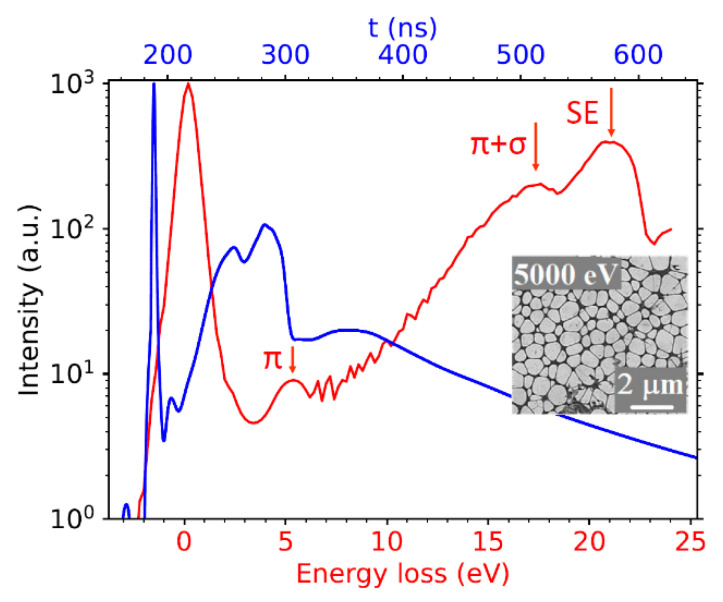
As-measured ToF spectrum (blue) of 1 ML graphene layer. Converted energy-loss spectrum (red) contains graphene-related plasmons. Inset: SLEEM image of graphene sheets.

The transmission signal proved itself sufficiently high even for ultra-low energies down to 25 eV, as shown in Figure 7. The figure illustrates the ability of our SLEEM/ToF device to measure spectra at such low energies. The low energy peak near 5 eV and the peak rising from a “background” of secondary electrons at 17 eV are interpreted as the π and (π + σ)-plasmon peaks. The latter, partly hidden in the signal of secondary electrons, culminates at a loss of approximately 21 eV.

**Figure 8:**
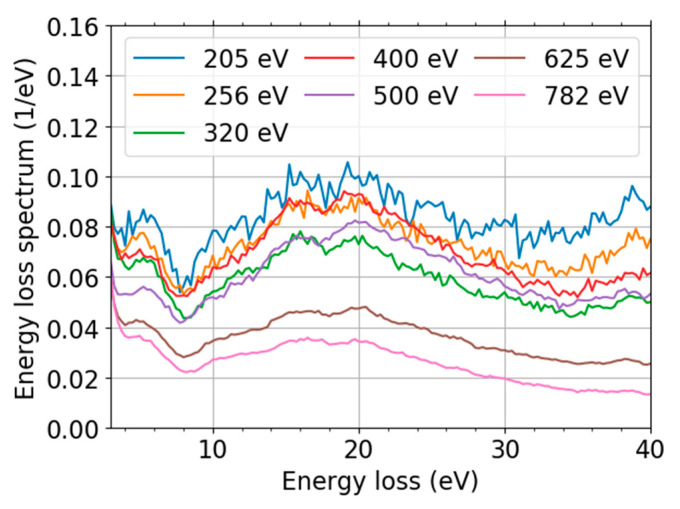
ToF energy-domain spectra with similar values of momentum transfer for selected landing energies. The data are divided by both area of the ZLP and width of energy bin.

We focus on the low landing energy interval (200, 800) eV in the following analysis. This is motivated by overlap with existing IMFP data for comparison purposes. An additional benefit is the minimized influence of the secondary electrons. The experimental data are presented in Figure 8. The spectral de-convolution was not needed because the spectral features observed were already well separated from the zero-loss peak (ZLP).

**Figure 9:**
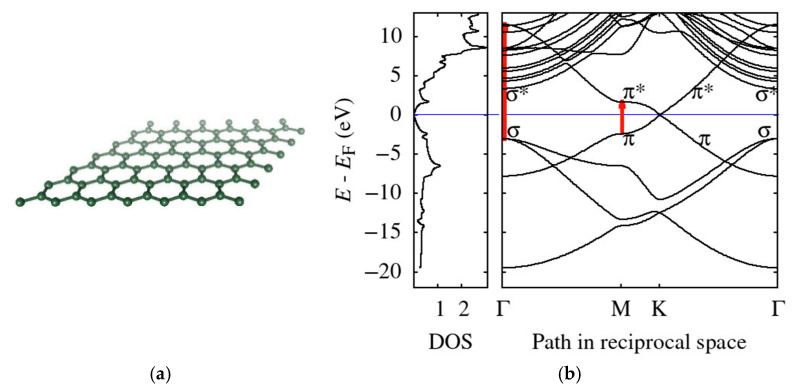
(**a**) The free-standing monolayer graphene structure produced by VESTA [62] and (**b**) theoretical DFT results for the total density of states (in states/eV atom) and energy-momentum dispersion relations for the electron bands with some of the important bands labeled accordingly. Vertical arrows (red) indicate transitions that correspond to energy losses of the plasmons. Both DOS and bands are referred to the Fermi energy *E_F_*.

In order to illustrate and to explain the origin of the two dominant loss features in the EELS spectra of graphene, Figure 9 provides theoretical results for both the DOS and the band structure. The DFT simulations presented in Figure 9 agree well with LDA results published in Refs. [59,60]. The Fermi velocity determined from the band structure at the K-point is approximately 0.9 × 10^6^ m/s (experimental value is 1.1 × 10^6^ m/s [61]). DFT-LDA is known to underestimate the experimental value of Fermi velocity, while the many-body GW simulations often provide better agreement with the experiment [60].

We used vertical arrows to mark two transitions corresponding to energy position of the two above-mentioned loss-peaks in Figure 9. Our corresponding DFT values are 4.1 eV (interband transitions: π → π* at M-point) and 14.6 eV (σ → π* at Г-point). The experimental values close to vanishing momentum transfer are 4.9 eV and 15.3 eV, respectively [63]. Both theoretical results, based on simple interband transitions, underestimate the experimental values. In fact, the experimental data are always measured for some finite non-zero momentum transfer shifting the loss features to higher energy losses (as clearly seen in data presented in Table 1), which contributes to this discrepancy. Moreover, the exchange correlation effects may blue-shift the plasmon by approximately 0.5 eV [63]. In [64] (Figure 2b), they directly measured the electronic transition in K-space, leading to the π + σ plasmon. While this was carried out on highly oriented pyrolytic graphite, it is nonetheless a confirmation of the red arrow in Figure 9b corresponding to the σ-σ* transition.

It is well known that the crudest independent-particle approximation (IPA) does not describe the graphene EELS spectra well [71]. The random-phase approximation (RPA), in which the model becomes more complex and the plasmons may mix several interband transitions, produces more accurate results. Moreover, it is a bit controversial to interpret the peaks in EELS spectra as interband electronic transitions and plasmons; see Ref. [72] and related references therein. Indeed, the spectrum is composed of single-particle and collective effects. Hence, a finer condition than a simple maximum of the energy-loss function (ELF), proportional to Im{-1/ε(*q*, *T*)} with ε being the dielectric function, is needed to distinguish between two kinds of effects—single-particle and collective—(here, *q* denotes the MT and *T* stands for the energy loss). The condition is found to be Re{*ε*(*q*, *T*)} = 0 [72].

**Figure 10:**
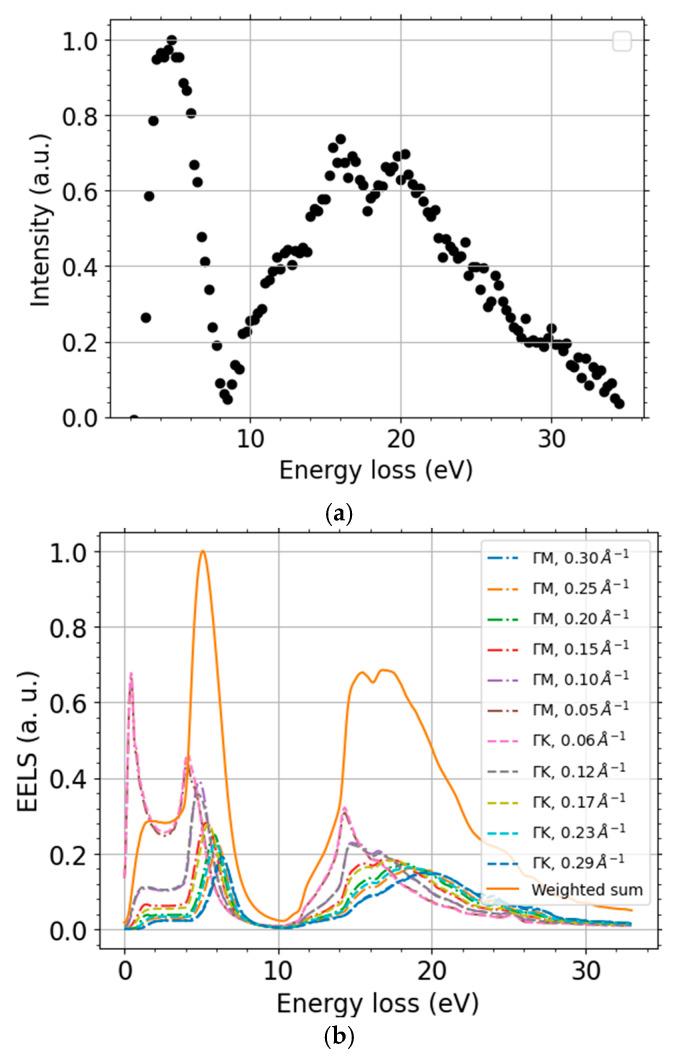
Scaled EELS spectra: the processed measurement data at landing energy 320 eV (**a**) and DFT-calculations (**b**). The DFT data calculated along the two paths ГM (dash-dotted line) and ГK (dashed line), both segments summed up to momentum transfer *q* = 0.3 Å^−1^ (solid line).

We calculated momentum-resolved EELS spectra within MBPT beside the already discussed DOS and band structure. They provide a deeper insight into the graphene spectra and allow us to corroborate the observed range of *q* and to compare these predictions with the measured spectra. The MBPT-EELS spectra along two segments in the reciprocal space, ГM and ГK, up to *q* ≈ 0.3 Å^−1^, are displayed in Figure 10b. Let us note that positions of both dominant loss-peaks from upon the simple interband transitions estimated from the DFT band structure, Figure 9b, correspond well to the more accurate RPA calculations close to the Г-point (Figure 10b, data for the lowest MT). The positions of maxima of the two main loss features are 4.1 eV and 14.3 eV for *q* = 0.055 Å^−1^ in the DFT-EELS spectra. The multiple simulation data-sets displayed in Figure 10b clearly show the dispersion relation of the two loss-peaks.

Our MT-resolved simulations agree well with other calculations [71,73] and experiments [63,67]. The selected maximum value of MT is independently supported by theoretical tracing of the escaping electrons by means of in-house-made scripts. We intend to publish the full theoretical spectra and their detailed analysis, including the calculation of the IMFP from the MBPT-EELS elsewhere. We calculated the above-mentioned weights simply as a product of areas of semi-annuli with each semi-annulus containing a single computed MT *q* and approximated Lorentz factor, yielding “*q dq*/(*q*^2^ + *q*_E_^2^) → *dq*/*q*” for *q* ≠ 0 and 0 if *q* = 0 ≠ *q*_E_, not including the kinematic restrictions on *q*. The energy-loss *T* dependence is contained in *q*_E_ that is defined as *q*_E_(*T*, *E*_L_) = *T*/(*ħ v*(*E*_L_)), with the velocity *v* determined from the landing energy *E*_L_. Let us compare the MBPT-EELS intensity from the two segments, collected up to the maximum MT using a weighted sum, with the experimental data (both ZLP and baseline subtracted). Figure 10 shows that both positions of the simulated peaks and their intensity are in a good agreement with the processed experimental spectra. The slight discrepancy is due to the approximations used in the data processing and simulations. Moreover, these simulations support the idea that the splitting of the (π + σ)-plasmon is given by the weighted sum of the momentum-resolved (π + σ)-plasmon intensity over different MTs. This agreement gives us more confidence in the processed experimental data, and we regard them to be ready for the following processing to obtain IMFP.

**Figure 11:**
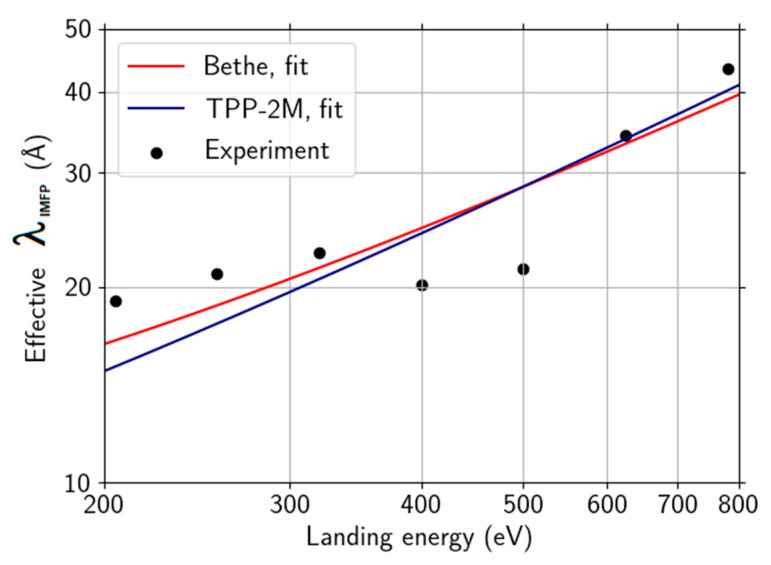
Effective IMFP calculated from the ToF energy-domain spectra in Figure 10 compared to effective IMFP derived according to TPP-2M and Bethe formulae.

Applying the log-ratio method on the straight-line segment baseline-corrected energy-loss spectra, we arrived at the IMFP values presented in Figure 11. We used theoretical “monolayer thickness” graphene (estimated from bilayer graphene) with *d* = 3.35 Å as the thickness of the sample in the log-ratio formula. The increase in the IMFP with increasing energy is a direct consequence of decreasing area of scaled loss-spectrum, as shown in Figure 8, and the log-ratio formula.

Let us compare our result with the data in Figure 5 in Ref. [74], displaying IMFP obtained from a data-driven model within the range of energies from close to zero up to 600 eV. Their IMFP values exhibit a peak within a range of (220, 270)eV approximately. Apart from the peak reaching slightly over 60 Å, their IMFP increases with oscillations on top of a fairly linear trend, ascending from about 20 Å at 200 eV up to 35 Å at 600 eV. Furthermore, the authors interpret the presence of the high peak as unreliability due to a strong signal in the C KVV Auger spectra peak of the (polycrystalline gold-supported) graphene. Inside the aforementioned energy range, our results agree well, apart from the strong peak in the data of Ref. [74], meaning both the values and the general trend agree, except for the strong peak in the data of Ref. [74]. Our effective IMFP data only exhibit a minor peak at slightly higher values of energy. Bethe equation $\lambda_{IMFP}\left(E\right)=E/\left[E_{P}{}^{2}\beta \;ln\left(\gamma E\right)\right]$ fit values to our data, β = 0.0116 eV^−1^Å^−1^, γ = 0.042 eV^−1^ compare well with the results in Ref. [74], β = 0.0098 eV^−1^Å^−1^, γ = 0.053 eV^−1^. The free-electron plasmon energy of carbon is fixed to *E_P_* = 22.3 eV.

## 4. Discussion

Let us begin the discussion by recalling some of the previously available free-standing monolayer graphene experiments and theoretical simulations. Two main material features dominate the graphene energy-loss spectra, namely the π and (π + σ) plasmon peaks. Their position (frequency ω_plasmon_ or energy-loss *T*_plasmon_) and width vary with momentum transfer *q*, due to the dispersion relation *T*_plasmon_(*q*), and number of layers *n*. The π-plasmon range is [4,12] eV and the (π + σ)-plasmon range is [13,30] eV, as shown in Table 1. Furthermore, existing experiments reveal that sample tilt affects the dispersion relation (Figure 4a in Ref. [67]).

A comparison of Figure 10 with the results in Table 1 reveals good agreement in the positions of both peaks. The wide spread of peak positions depending on MT, in both Figure 10b and Table 1, implies that knowledge of a range of MT values collected during experiments is highly desirable. An upgrade of the device is already planned in order to have more control over the MT realized. MBPT simulations may help to verify the achievement of the goal and/or aid in active corrections of the spectra for MT to provide a better estimate of the IMFP.

Variations of the peak positions with landing energies in Figure 8 are very small, so we can ascribe them to variations in MT. Variations in MT may stem from different settings of the active components in the UHV SLEEM/ToF device that affect the effective aperture size and local tilt of the not exactly planar sample.

The effective aperture size of the UHV SLEEM/ToF device contributes to a splitting of the (π + σ)-plasmon peak, since our measurements are composed of contributions of several values of MT. This can be seen from our MT-resolved DFT/MBPT simulations along the same path as the data in [63] (the full theoretical spectra will be published elsewhere). This is corroborated by superimposing the data of Ref. [63] (Figure 1 there) corresponding to different values of MT.

Tracing the electron trajectories after escaping the sample for energies close to the landing energy, i.e., close to zero energy loss, reveals two intervals of the acceptance angle, namely 0–5° and 28–32°. Moreover, the primary electron beam illuminating the sample is not parallel, and its convergence angle is magnified due to the field produced by the sample bias. This means that even though the condition of sufficient beam tilt as described in [75] may be not met, because of the beam convergence, it can be approached.

The contribution of secondary electrons (SEs) cannot be currently removed from the spectra without artificially cutting the spectra at some low energy loss. Measurements focused on SE suppression are already planned. The method to obtain the IMFP from the measured loss spectra, so-called “dielectric formalism”, consists of fitting parameters of a suitable model of dielectric function *ε* to the spectra. The IMFP is then calculated via a double integral of a longitudinal differential inelastic cross-section formula which is proportional to the energy-loss function Im{1/ε}. The method is described, e.g., in [76] and it also assumes a homogeneous sample to derive a relation between the spectra and the dielectric function *ε*. Since the sample examined here is crystalline, the above method should be applied with caution. Recently, another group determined an IMFP-like quantity using the reflectivity and transmissivity of a graphene sample [12] but not using the ToF spectra.

## 5. Conclusions

The above-discussed electron energy-loss spectra of free-standing monolayer graphene transmitted with very slow electrons agree well with theoretical simulations and existing literature, thus corroborating the functionality of the UHV SLEEM/ToF device. This applies to a wide range of landing energies from 3500 eV down to 25 eV.

One of the main benefits of this device is the possibility of using free-standing ultra-thin samples. This enables one to eliminate substrate effects and to avoid multiple inelastic scattering. As a result, the EELS data analysis is significantly simplified. The energy resolution of the ToF spectrometer, 0.5 eV at the landing energy of 50 eV, is more than acceptable for study of the graphene sample, as presented here.

Furthermore, our results regarding the energy dependence of the effective IMFP are in good agreement with other published EELS spectra of graphene. The study will be continued on a variety of 2D materials illuminated with extremely slow electrons with foreseen applications to nanoelectronics.

## Figures and Tables

**Table 1:** An overview of various plasmon dispersion relation *T*(*q*) (eV) data in the literature, experimental (Exp.) and simulated (Sim). Positions of the plasmon features *T*(*q*) (eV) and range of their variations with the measured momentum transfer *q* (Å^−1^), 2nd interval. Dash indicates a value impossible to obtain from the energy-loss range provided in the data.

Reference (Exp.) or (Sim.)	π-Plasmon *T*(*q*) (eV), *q* (Å^−1^)	(π + σ)-Plasmon *T*(*q*) (eV), *q* (Å^−1^)
[65] (Exp.)	[4.9, 6.6], [0.05, 0.38]	-,-
[66] (Exp.)	[4.9, 8.6], [0.0, 0.7]	-,-
[67] (Exp.)	[4.0, 12.3], [0.0, 1.56]	[13.5, 30], [0.0, 1.21]
[63] (Exp.)	[4.8, ≈10], [0.0, 1.4]	[15.3, ≈30], [0.0, 1.4]
[68] (Sim.)	[4.8, 7.3], [0.03, 1.7]	-,-
[69] (Sim.)	[≈4, ≈12.5], [≈0.0, 1.4]	[≈14.5, -], [≈0.0, 1.4]
[63] (Sim.)	[≈4, ≈10], [0.0, 1.3]	[≈14, ≈33], [0.0, 1.3]
[70] (Sim.)	[4.3, ≈13], [≈0.0, 1.7]	[≈13.9, -], [≈0.0, 1.7]

## Data Availability

Data sharing not applicable.
